# Automated Data Quality Control in FDOPA brain PET Imaging using Deep Learning

**DOI:** 10.1016/j.cmpb.2021.106239

**Published:** 2021-09

**Authors:** Antonella D. Pontoriero, Giovanna Nordio, Rubaida Easmin, Alessio Giacomel, Barbara Santangelo, Sameer Jahuar, Ilaria Bonoldi, Maria Rogdaki, Federico Turkheimer, Oliver Howes, Mattia Veronese

**Affiliations:** a Department of Neuroimaging, Institute of Psychiatry, Psychology & Neuroscience, King's College London, London, United Kingdom; b Department of Psychosis Studies, Institute of Psychiatry, Psychology & Neuroscience, King's College London, London, United Kingdom; c Department of Psychological Medicine, Institute of Psychiatry, Psychology & Neuroscience, King's College London, London, United Kingdom; d H. Lundbeck UK, Ottiliavej 9 2500 Valby, Denmark; e Institute of Clinical Sciences (ICS), Faculty of Medicine, Imperial College London, Du Cane Road, London W12 0NN; f Department of Information Engineering, University of Padua, Padua, Italy

**Keywords:** FDOPA, PET, quality control, QC, convolutional neural networks

## Abstract

*Introduction.* With biomedical imaging research increasingly using large datasets, it becomes critical to find operator-free methods to quality control the data collected and the associated analysis. Attempts to use artificial intelligence (AI) to perform automated quality control (QC) for both single-site and multi-site datasets have been explored in some neuroimaging techniques (e.g. EEG or MRI), although these methods struggle to find replication in other domains. The aim of this study is to test the feasibility of an automated QC pipeline for brain [^18^F]-FDOPA PET imaging as a biomarker for the dopamine system.

*Methods.* Two different Convolutional Neural Networks (CNNs) were used and combined to assess spatial misalignment to a standard template and the signal-to-noise ratio (SNR) relative to 200 static [^18^F]-FDOPA PET images that had been manually quality controlled from three different PET/CT scanners. The scans were combined with an additional 400 scans, in which misalignment (200 scans) and low SNR (200 scans) were simulated. A cross-validation was performed, where 80% of the data were used for training and 20% for validation. Two additional datasets of [^18^F]-FDOPA PET images (50 and 100 scans respectively with at least 80% of good quality images) were used for out-of-sample validation.

*Results*. The CNN performance was excellent in the training dataset (accuracy for motion: 0.86 ± 0.01, accuracy for SNR: 0.69 ± 0.01), leading to 100% accurate QC classification when applied to the two out-of-sample datasets. Data dimensionality reduction affected the generalizability of the CNNs, especially when the classifiers were applied to the out-of-sample data from 3D to 1D datasets.

*Conclusions*. This feasibility study shows that it is possible to perform automatic QC of [^18^F]-FDOPA PET imaging with CNNs. The approach has the potential to be extended to other PET tracers in both brain and non-brain applications, but it is dependent on the availability of large datasets necessary for the algorithm training.

## Introduction

Novel and cheaper technologies have allowed the collection, storage and use of large neuroimaging datasets that are now being acquired at a very high pace [1]. This big data “revolution” has enabled the use of novel data-driven analytical techniques to tackle unanswered questions about the brain in normal and pathological conditions [2]. However, high noise levels, missing or incomplete data, motion artefacts, and scanning equipment miss-calibration might lead to poor data quality and inconsistent results [3]. While periodic inspections are necessary to maintain the proper functioning of medical imaging acquisition systems [4], [5], image quality control (QC) is required to guarantee reliable and accurate analyses [6], [7], [8]. Moreover, the complexity of a neuroimaging study, in which the relationship between research sponsors and acquisition centres can be mediated by third part organisations (e.g. CRO or universities), makes the QC process even more critical as the objective and qualitative assessment of the acquired images is included in the contractual terms for the scan's payment.

Currently, visual inspection is the common QC method adopted for the majority of neuroimaging modalities. Images are visually examined by experts and discarded if either raw data or analysis outputs do not comply with pre-defined standards. This includes the extraction of image-derived features (also known as “image-derived phenotypes” or IDPs), and their comparisons with a range of normal and biologically plausible values [9]. However, manual or semi-automated image assessment, can lead to systematic biases arising from subjective judgements of the readers. This is true for any type of medical imaging modality, including brain imaging [10]. Moreover, in large datasets, visual assessments are not practical. An example of this is represented by UK Biobank and its 100,000 neuroimaging scans, which make QC via visual inspection unfeasible [11]. Additionally, even when manual QC is possible, image artefacts arising from wrong acquisition parameters might be missed by human operators [12] and can pose threats that are difficult to foresee before the full completion of a study. This is well-known in MRI imaging, as poorly executed QC can compromise the trustworthiness of its scientific findings [13].

**Fig. 1:**
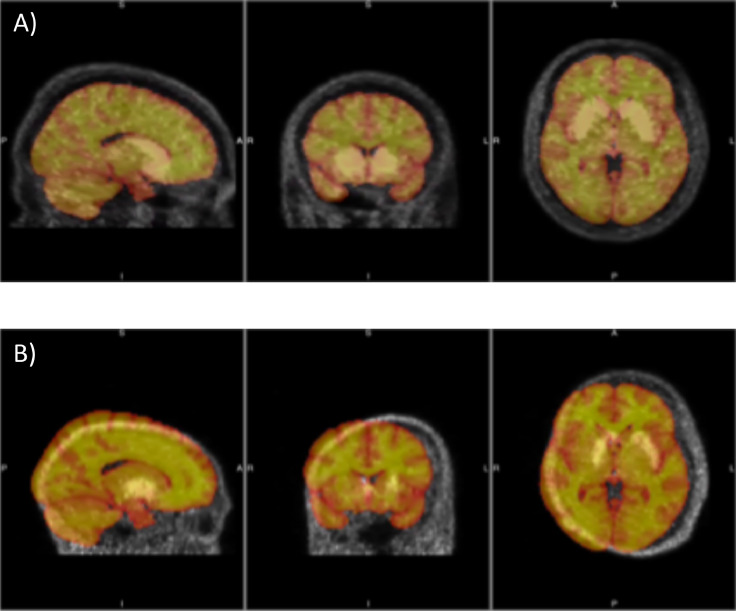
w Misalignment of FDOPA PET images to an MRI template. Example of aligned (a) and misaligned (b) [18F]-FDOPA PET images with an MRI template overlapped (orange). Sagittal (left), coronal (middle), axial al (right) views are shown.

A potential solution to this problem is to use automated QC protocols tailored to the specific characteristics of the data collected. Several studies have reported the use of automated QC in neuroimaging. In EEG, computer-based methods are normally applied for the removal of artefact rejections (e.g. muscle and eye movements, or electrode displacements) [14] from which it is possible to obtain information on the quality of the acquired data. In MRI, both Deep Learning (DL) and Image Quality Metrics (IQMs) have been used for single-site and multi-site datasets [15], [16], [17]. While IQMs require to predefine a priori the features to be used for the QC assessment, DL methods have the great advantage of extracting them automatically in order to optimise the performance of the task [18]. Resonance frequency, Signal-to-Noise Ratio (SNR), image uniformity, spatial linearity, spatial resolution, slice thickness, slice position/separation, and phase related image artefacts have been used as IQMs [17]. IQMs have also been used to perform QC in PET images [19]. In this case, similarity indexes measuring the goodness of image co-registration between PET and the corresponding structural MR images have been used. No automated QC method using DL for PET currently exists. Particularly, in PET imaging, trade-offs between resolution, noise and quantitative accuracy of the measurements might represent a challenge for automated QC [20]: a PET scan requires expertise from different people involved for tracer synthesis, experimental protocol design, reconstruction and analysis, which leads to a high variability in final image quality, as methods may vary. Despite these challenges, this study attempts to test and validate a DL automated QC method for brain PET imaging, considering [^18^F]-FDOPA PET (hereinafter FDOPA) imaging as a case study (Fig. 1).

FDOPA PET has been used for over 35 years to study dopaminergic function in the living human brain [21]. The accumulation of FDOPA in the brain reveals the functional integrity of the presynaptic dopaminergic synthesis. Multiple studies have shown how the FDOPA PET can be used to find abnormalities in dopaminergic transmission that occur in brain disorders, such as Parkinson's Disease (PD) [22], schizophrenia [23] and in certain types of tumours [24]. With FDOPA PET applications gradually expanding, imaging datasets are becoming larger, making it an ideal testing case for automated QC algorithms. This paper will take advantage of a unique FDOPA PET dataset of scans (number of scans: 715, number of individuals: 594) acquired with different PET scanners and imaging protocols and which have been through manual QC. This allows the generalisability of results across sites and conditions, as well as the method performances with data dimensionality reduction, to be examined.

## Methods

### DenseNet

A widely used DL method is represented by Convolutional Neural Networks (CNNs). CNNs are a type of neural networks that allow the processing of data with a grid-like topology (like images), automatically extracting data features depending on the ultimate task one aims to accomplish (in this particular work the classification of the image quality). In general, CNNs are made of blocks composed of several layers. In each layer, images are convoluted to return feature maps that could be better used to carry out the classification work. Depending on the CNN considered, at the end of each block or even layer, the size of the feature maps is reduced. This process can be repeated several times (by adding more layers and/or blocks) in order to obtain new feature maps that can be filtered again until the most relevant features of the image are found. Finally, the values of the last feature maps are concatenated into a vector which is the input of the last block within the CNN. Linear functions are applied to the final vectors to return a new vector including as many elements as the possible classification classes. Each element is a value between 0 and 1 representing the probability of the image to belong to that class [25].

CNNs have shown to achieve state-of-the-art performances on various medical imaging tasks, including image classification, image segmentation, object detection and automated image analysis, thanks to their self-learning ability [26], [27], [28]. In the recent study from Küstner T. et al., CNNs have been showcased for the assessment of medical image quality, in particular for automated detection of motion artefacts in magnetic resonance imaging [29].

Among the different existing CNNs, one that has been increasingly applied to tasks in the medical imaging field is DenseNet [30], [31], [32], [33]. Due to the outperforming results that it has shown when compared to other networks [34], DenseNet was adopted in this study to perform QC. The method was implemented using a supervised learning approach, in which the CNN training was based on labelled data (e.g. aligned/misaligned, good SNR/poor SNR) derived from the manual QC.

DenseNet connects all layers with matching feature-map sizes directly with each other. To preserve the feed-forward architecture, each layer obtains additional inputs from all preceding layers and passes its own feature-maps to all subsequent layers. Contrary to other networks such as ResNets [35], features are not summed but concatenated before passing into a layer. Due to this dense connectivity pattern, DenseNet models are characterised by fewer parameters than traditional CNNs, as there is no need to learn several times the same redundant feature-maps. In this way, DenseNet alleviates the vanishing-gradient problem, and strengthens feature propagation with feature reuse while reducing the number of parameters learnt. DenseNets have returned significant performances when trained on the most commonly used datasets available online, while requiring less computation to achieve high performances, comparable to other state-of-the-art CNNs [34].

Given an image *x*_0_ (defined as the input of a CNN with *L* layers) the *n^th^* layer is defined as:

$$x_{n}=H_{n}\left(\left[x_{0},\;\ldots ,\;x_{n-1}\right]\right)$$

with [*x*_0_, …,  *x*_*n* − 1_] being the concatenation of the feature maps of layers 0,  …,  *n* − 1, and *H_n_* being the *n^th^* non-linear transformation. *H_n_* is implemented as a composite function of three operations: Batch Normalisation (BN – transformation that maintains the mean of the map close to 0 and the standard deviation close to 1), Rectified Linear Unit (ReLU - the activation function that takes as inputs the feature maps and returns either zero or the same values if positive) and convolution with a [4*x*4] convolution kernel. These operations (BN-ReLU-Convolution) are applied at each layer in the block to extract the features, while maintaining the same size of the feature maps as these are concatenated to those output of the subsequent layers. In order to perform down-sampling, a convolution and pooling layer are added at the end of each block, forming the so-called transition layers and allowing the feature maps to be reduced in size.

Given *k* feature maps generated by *H_n_* at each layer, *k*_0_ number of channels of the input, the *n^th^* layer is characterised by *k*_0_ + *k* ×  (*n* − 1) feature maps. *k* is a hyperparameter that can be tuned before training, called *growth rate*.

The number of feature maps can be reduced in two ways: the first way is to use a so-called bottleneck layer which is an extra [1  ×  1] convolutional layer added before each [4  ×  4] convolutional layer, while the second is to reduce them at the transition layers. This is done by multiplying the number of feature maps by a compression factor, θ (0 < θ ≤ 1; when θ = 1 no compression occurs, and the number of features is not reduced).

Both approaches can increase the computational efficiency of the network since less feature maps will be produced. Other settings that help to prevent overfitting include dropout rate and regularisation parameters [34]. Dropout rate is a predefined probability at which, in a certain layer (dropout layer), the output vectors are removed from intermediate steps. Regularisation parameters, such as *L*_1_ (sum of weights) or *L*_2_ norm (sum of squared weights), can be added as a penalty to each layer in order to penalise large weights that might result from overfitting the data.

**Fig. 2:**
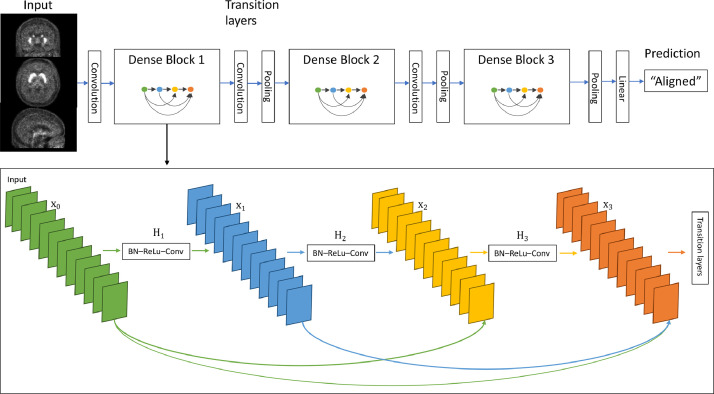
DenseNet architecture. Architecture of the implemented DenseNet with three dense blocks, each of which including four layers and growth rate *k* = 12 (adapted from Huang G. et al.34 Convolution is applied to the 3D input image, in order to create the feature maps to be used as input for the first block. Once entered the block, the feature maps are filtered (they are normalised and fewer most relevant features are extracted). These are then used as input for all the other layers within the block and concatenated together. Once exited the block, convolution and pooling operations are applied to reduce the size of the resulting feature maps. These processes are repeated until the feature maps (smaller in size, due the pooling layer at the end of each block) reach the final linear layer where they are converted into vectors of most relevant features that will lead to a final prediction based on the images belonging to the validation dataset. The final prediction is represented by the probability of each image in the dataset to belong to a given set of classes (in this example derived from CNN1, the probability of being aligned or misaligned). The class with the greater value associated to it will be the final prediction of the network to the input image.

Fig. 2 shows an example of the architecture of a DenseNet with three dense blocks, each of which includes four layers and growth rate *k* = 12. No bottleneck or compression were considered.

### Datasets

**Fig. 3:**
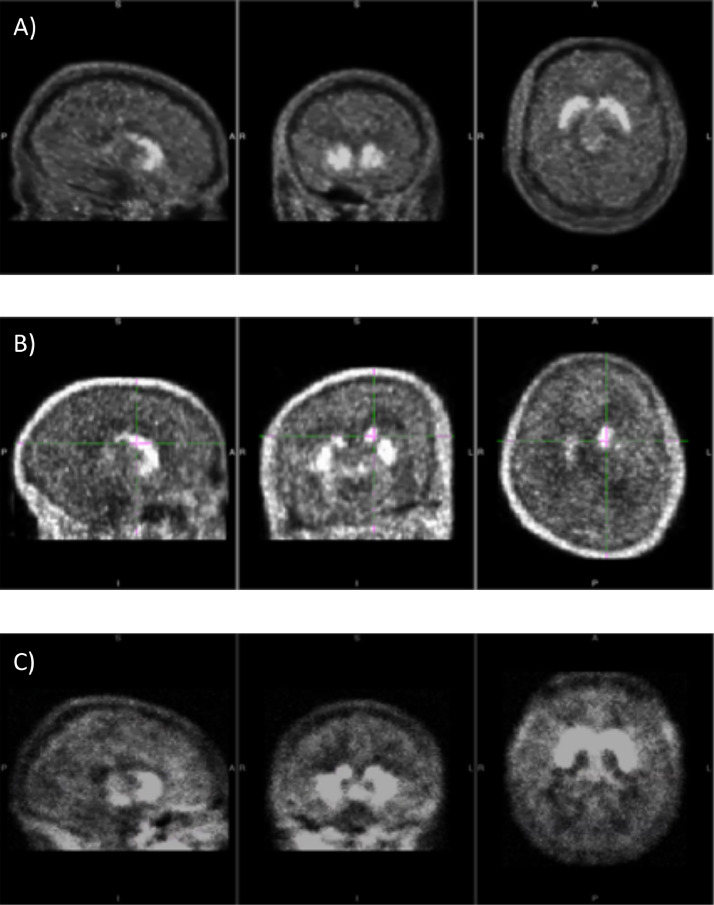
Examples of FDOPA maps. Examples of a good quality FDOPA map (a), misaligned FDOPA map (b), and noisy FDOPA map (c) from Dataset 1.

Three different datasets were used for training and testing (Table 1). *Dataset 1* consists of 200 good-quality visually inspected FDOPA maps (Fig. 3***a***), 200 misaligned FDOPA maps derived from PET scans prior to motion correction (Fig. 3***b***) and 200 noisy FDOPA maps simulated by adding white random noise (Gaussian distribution with zero mean and Standard Deviation (SD) equal to 20% of noise-free voxel value) to the good quality FDOPA maps (Fig. 3***c***). This level of noise was simulated by analysing the SNR distribution of historical data from our internal PET neuroimaging database (NODE [36]).

**Table 1:** Datasets used in the study for training and testing.

	Total N of scans	QC-passed scans	QC-failed scans	Training	Testing
Dataset 1	400	200 (measured data)	200 (simulated data)	Yes	Yes Implementing cross-validation model (20% of the data were randomly removed from the training sample and used for testing only)
Dataset 2	50	39 (measured data)	11 (measured data)	No	Yes
Dataset 3	100	90 (measured data)	10 (measured data)	No	Yes

The original maps in *Dataset 1* were acquired using three different PET scanners: Siemens Biograph 6 HiRez (Siemens, Erlangen, Germany), Siemens/CTI ECAT HR+ 962 (Siemens, Erlangen, Germany) and Siemens Biograph TruePoint 6 CT45544 (Siemens, Erlangen, Germany). The three scanners have similar spatial resolution (4.5 ± 0.24mm, 4.8 ± 0.2mm and 4.5 ± 0.2mm, respectively) and comparable sensitivity (4.2 cps/kBq, 4.2 cps/kBq and 4.5 cps/kBq, respectively).

*Dataset 2* consists of 50 maps acquired using the scanner Siemens Biograph 6 HiRez (Siemens, Erlangen, Germany), as in *Dataset 1*. Among the 50 maps of *Dataset 2*, 11 are misaligned FDOPA maps and 39 are FDOPA aligned maps and normalised in MNI space. *Dataset 3* comprises 100 maps acquired in six different scanning sites, which are all independent from those mentioned for *Dataset 1* and *Dataset 2*. This dataset includes 10 misaligned FDOPA maps and 90 FDOPA maps aligned and normalised in MNI space. All the maps have acceptable SNR level as determined by visual inspection. In *Dataset 3* the scanners used were Siemens Biograph TruePoint PET/40 CT, Siemens Biograph TruePoint PET/64 CT and Siemens/CTI ECAT HR+962.

All the FDOPA maps use the standardised uptake value ratio (SUVr), a simplified index of FDOPA uptake, defined as the ratio of the tracer activity in the striatum to that of the cerebellum acquired between 60 to 75 minutes [37]. This index is very similar to those used in simplified PET acquisition for clinical PET routine in oncological or neurological studies. The SUVr maps were all derived from dynamic PET acquisition accordingly to the same experimental protocol [38], [39] with a target injected radioactivity of ~150 ± 12 MBq (mean± SD). The maps with acceptable image quality were all manually assessed by an expert PET image analyst (MV or BS) and met all the following criteria: 1) plausible FDOPA PET signal distribution (identified by visual inspection), where the areas with highest PET uptake match anatomical regions with highest dopamine content, 2) max between frame motion realignment <8mm (as derived by between-frame image realignment), 3) adequate anatomical atlas co-registration (identified by manually checking the striatal and cerebellar anatomical masks on individual FDOPA PET summed image), 4) physiological range of SUVr values in striatum (SUVr>1.5) and cerebellum (SUVr~1) and 5) suitable spatial normalisation of the individual SUVr FDOPA map into standard space (identified by visual inspection).

### CNN implementation

Implementation of the CNN DenseNet to the FDOPA PET QC problem required some preliminary tuning. Training was performed to assess the number of epochs (number of iterations to update the weights) to consider, with the highest number considered being 10,000. Similarly, the number of layers was investigated. First attempts tested a high number of layers (121), in lines with previous publications training DenseNet. Finally, three dense blocks with 4 layers were implemented; and a growth rate of 12 (*k* = 12) was used with no compression or bottleneck used when training the network.

Two optimization methods, an adaptive moment estimation (ADAM) [40] and stochastic gradient descent (SGD) [41], were initially tested to assess which one performed best to use for the final training. They both adopted batch size of 32; this is the hyperparameter representing the number of training samples randomly chosen, utilised to make the prediction at each iteration. The learning rate used for testing both optimisers was set to 10^−3^. SGD was set with additional parameters: momentum equal to 0.9 (to accelerate the gradient minimisation in the relevant direction) and Nesterov momentum (to average the direction of the gradient over multiple time steps). Default parameters were used in Adam [40]. L_2_ regularisation and a dropout rate equal to 0.2 were chosen to prevent overfitting [34].

Given the parameters outlined above, two neural networks were trained for two different tasks: to assess the misalignment from a standard reference space and to identify if the SNR level was within predefined acceptable noise level ranges. The first network (CNN1) assessed whether the input map is aligned to a standard FDOPA template Montreal Neurological Institute standard space (matrix dimension: 91  ×  109  ×  91; voxel size: 2mm isotropic). Spatial normalisation into MNI space is not part of the data acquisition itself but it represents one of the pre-processing steps performed to compare the scans with normative values. The other network (CNN2) aimed to discard maps whose SNR were outside acceptable noise level ranges. Both CNN1 and CNN2 were trained on a total of 400 maps (200 maps with acceptable quality and 200 maps simulated to fail manual QC).

**Fig. 4:**
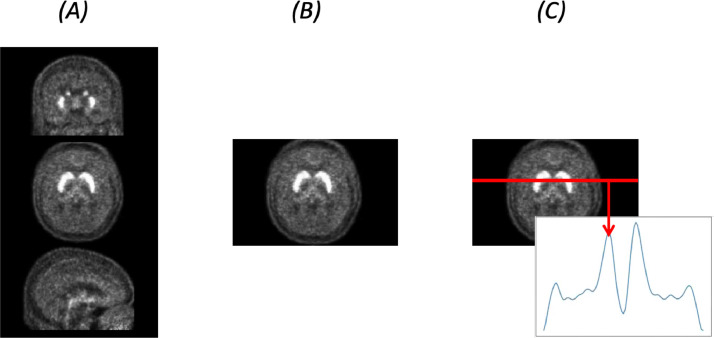
Dimensionality of the datasets. Representative images for the 3D dataset (a); 2D dataset (b); and 1D dataset (c). 2D maps are obtained by sampling a central axial slice from the 3D images through the striatal region. 1D datasets are obtained by tracing a representative line on the 2D maps through the striatal region.

The same models were trained on the original three-dimensional (3D) maps (matrix dimensions: 91  ×  109  ×  91), two-dimensional (2D) datasets comprising the middle single slices of the 3D maps where the striatum uptake is visible (matrix dimensions: 91  ×  109  ×  1), and on one-dimensional (1D) datasets (matrix dimensions: 91  ×  1  ×  1), obtained by tracing a representative line of the 2D slice (Fig. 4). This was possible by keeping the same parameters for each CNN and only changing the dimensions of the Convolutional and Pooling layers according to the dataset considered.

Training was performed by using the TensorFlow 2.3 library in Python (Python version 3.7). Computations were run with three Central Processing Units (CPUs) with 18GB of memory.

### Experimental design and Statistical analysis

Dataset 1 was used for test in-sample. Cross-validation was performed using 5 subsets during training with a balanced random validation set including 20% of the data of the entire dataset. Consistently with pattern recognition classification nomenclature [42], results obtained from training were evaluated based on the categorical accuracy, precision, recall and AUC and cross-entropy as a loss. Accuracy was computed as the total number of QC'ed images correctly labelled (i.e. number of true positives and true negatives) over the size of the dataset. Note that an accuracy equal to 1 would correspond to perfect classification, while an accuracy equal to 0.5 would correspond to a classifier randomly QCing the images. Precision (in other works refer as positive predictive value) was calculated by simply dividing the number of true positives by the sum of total positives (true and false). Recall (in other works refer as sensitivity) was computed as the total number of true positives divided by the sum of true positives and false negatives. AUC was computed by considering the ROC-curve. Finally, the cross-entropy was calculated as − (y_i_ log(p_i_) + (1  −  y_i_) log(1 −  p_i_)), where i is the sample considered, y_i_ is the correct binary value indicating whether the sample is a good- or poor-quality map and p_i_ is the binary value indicating whether the sample has been labelled as a good- or poor-quality map. A value of cross-entropy equal to 0 would mean perfect classification.

The five cross-validated models trained on Dataset 1 were finally tested on Dataset 2 and Dataset 3 to assess their overall performances on completely new datasets. To note that the data came from the same scanning sites (Dataset 2) and independent scanning sites (Dataset 3), as compared to the data used for training. Same performance indexes were used to investigate the capacity of CNNs to perform a correct QC of the data.

## Results

### DenseNet optimisation

Preliminary trainings had allowed to tune some parameters such as the number of epochs and layers. The highest number of epochs considered was 10,000, but stability was reached at epoch 300 several times after which CNN classification performances did not change (variation on accuracy and loss <1%). This number was hence chosen as limit for final trainings.

**Fig. 5:**
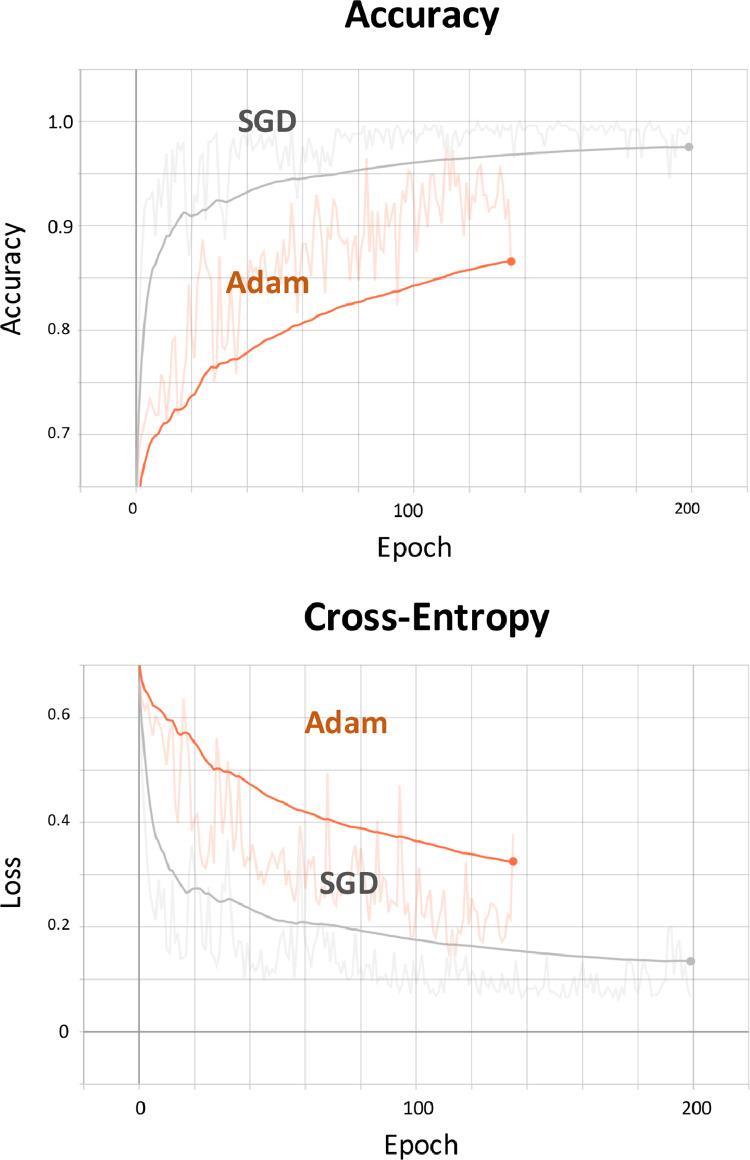
DenseNet optimisation. Performances of SGD vs Adam optimisers.

When choosing the number of layers for the final training, a high number (121) was initially considered. However, this resulted in data overfitting. The final training was performed with 4 layers. Finally, SGD was selected as the optimiser given its superior performances as compared to Adam [43] (Fig. 5).

### Method performance

**Fig. 6:**
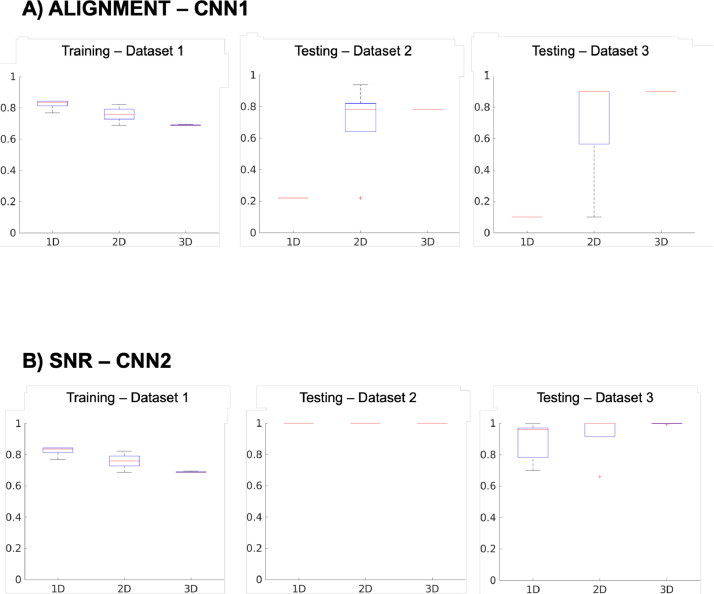
Algorithm performance. Boxplots showing (a) performances of CNN1 to test for alignment, (b) CNN2 to test for the level of SNR (b) – trained on 1D, 2D and 3D datasets. The y-axis shows the value of accuracy [0-1], whereas the x-axis shows the model considered.

A summary of the algorithm performance obtained when doing cross-validation with *Dataset 1* and testing out-of-sample with *Dataset 2* and *Dataset* 3 is reported in Fig. 6***a*** and Fig. 6***b*** *respectively*, while full statistics are reported in Tables 2-4.

**Table 2:** CNN performances with training dataset (in-sample test).

		Accuracy	Precision	Recall	AUC	Loss
CNN1	1D	0.90 ± 0.01	0.90 ± 0.01	0.90 ± 0.01	0.96 ± 0.01	0.20 ± 0.02
	2D	0.86 ± 0.04	0.86 ± 0.04	0.86 ± 0.04	0.92 ± 0.03	3.31 ± 2.27
	3D	0.86 ± 0.01	0.86 ± 0.01	0.86 ± 0.01	0.92 ± 0.01	1.33 ± 0.80
CNN2	1D	0.82 ± 0.01	0.82 ± 0.01	0.82 ± 0.01	0.88 ± 0.01	0.51 ± 0.13
	2D	0.76 ± 0.05	0.76 ± 0.05	0.76 ± 0.05	0.82 ± 0.05	0.61 ± 0.11
	3D	0.69 ± 0.01	0.69 ± 0.01	0.69 ± 0.01	0.74 ± 0.01	1.11 ± 0.53

In the training dataset (Dataset 1), the CNNs performances are very good, with the accuracy for the 3D models equal to 0.86 ± 0.01 (CNN1) for misalignment and 0.69 ± 0.01 for SNR (CNN2). These values show that variability of performance is consistent across the five cross-validated trained models for both networks (<2%).

**Fig. 7:**
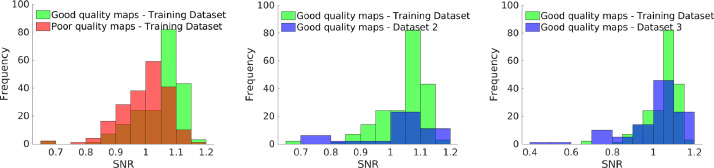
Distribution of image SNR for the three datasets used. Distributions of image SNR for Dataset1 (left), Dataset 2 (middle) and Dataset 3 (right). Green distributions refer to the FDOPA maps with good SNR used for the training. Red distribution refers to the FDOPA maps with poor SNR used for the training. Blue distributions refer to the SNR for FDOPA maps included in Dataset 2 and 3. SNR is defined as the mean of the brain FDOPA SUVR divided by its standard deviation.

When considering the out-of-sample datasets (Dataset 2 and 3), CNN performances improve even further as false negatives are not found in any of the 3D models (Table 3 and 4). These results are explained by the prevalence of poor QC cases (10% for misalignment and 0% for low SNR cases). Fig. 7 shows the distributions of the SNRs of the images from the three datasets: the noise levels for all good FDOPA images overlap irrespective from the acquisition scanners. The fact that the SNR distribution for the validation's datasets overlay with the one from the training, supports the good accuracy performance of CNN2 for both *Dataset 2* and *3*.

When reducing the dimensionality of the training dataset, the QC assessment performances (both in 2D and 1D models) overcome the results obtained with the 3D models (Table 2). However, when testing out-of-sample the lower dimensional models perform much worse than the corresponding 3D ones (Table 3 and 4). In CNN1 (misalignment), 2D model shows a loss of accuracy of 30% (Dataset 2) and 20% (Dataset 3), while the 1D model shows a loss of accuracy of 78% and 80%, respectively. In CNN2 (SNR), losses of accuracy are mainly reported in Dataset 3, corresponding to -6% for the 2D model, and -11% in the 1D model, respectively.

## Discussion

In this study a densely convolutional neural network approach was investigated to perform automated quality control for FDOPA brain PET imaging. The proposed CNNs assessed the misalignment of individual FDOPA PET images from a standard reference space and identified their SNR level. Good performances were shown both in the training dataset and in independent tests, while the accuracy worsened when reducing the dimensionality of the images.

### Test in-sample

Testing in-sample with 3D images gave good performances (accuracy: 0.86 ± 0.01, AUC: 0.92 ± 0.01), comparable to those obtained from automated QC tests on MRI data (accuracy: 0.83 ± 0.03, AUC: 0.87 ± 0.04) [15]. Comparing the two networks, the test for spatial alignment performed slightly better than the test on SNR. There are many reasons behind this performance. First of all, the assessment of the noise content of the FDOPA PET images was performed by visual inspection, simply comparing the contrast between striatal and non-striatal activity, as well as between brain signal and image background. This analysis is quite qualitative, but it is difficult to determine a simple SNR threshold applicable to any FDOPA PET scan below which an image can be rejected. In fact, in addition to the noise level, one should account for the noise spatial distribution: in a brain image like FDOPA PET, the striatal dopamine signal is the main target while the rest of the brain parenchyma is generally (but not always) secondary. Therefore, if the background signal is corrupted but the quality of the dopamine-rich regions preserved, the map can be still used for individual assessment. In such respect, multivariate classifiers would be more appropriate than a threshold-based statistics [44]. In terms of spatial alignment, instead, it is very common both in MRI and PET analysis to use motion limits (generally comparable with the resolution of the image, around 5 to 8 mm) above which the image is discarded. In such respect, image alignment is an easier test to be performed as compared to the SNR, even for a manual operator.

Secondly, SNR is a property of an image that can be manipulated in post-processing, and different denoising techniques have been proposed to restore the image quality of nuclear medicine scans when acquired with low counts. There is a growing interest in reducing the injected dose and the acquisition time, with the former that would lessen the potential risks of ionizing radiation and the latter to increase patient throughput [45], [46], [47], [48]. In contrast, artefacts due to motion are more difficult to be solved in post-processing especially when data are acquired in clinical setting.

### Test out-of-sample

The key question of the paper was to establish whether the performance of the DL networks for QC assessment could be replicated in independent datasets. For this specific purpose, we considered two sets of data using FDOPA PET maps acquired with the same and different scanners, as compared to those used in the training set. Moreover, these datasets used a prevalence of good quality FDOPA maps, which is consistent with real scenarios, in which only a small fraction of the data (~10%) is corrupted by misalignment and even a smaller fraction (<1%) from low SNR level. While the misalignment is generally caused by participant motion during the acquisition, the low SNR level might have more technical reasons, including a low injected dose leading to low counts or problem with the image reconstruction algorithm used. Nevertheless, both CNNs were able to preserve if not to improve their classification accuracy when tested in the two independent datasets (3D images). These results are encouraging since they potentially support the use of our method with newly acquired data. To note that misalignment was the only possible cause of QC failure for both *Dataset 2* and *3*, as all the FDOPA PET scans had a high SNR. This aspect needs to be considered, as it explains the excellent performance for the CNN2. In particular, the network performance could not be affected by false positives since there were no poor QC cases that could be misclassified.

### Reducing dimensionality

When considering how the models performed with respect to the data dimensionality, lower dimensional models (1D and 2D) showed higher performances and lower loss than models applied to 3D images in training. This result might suggest that lower dimensional datasets could be a solution to improve the computational efficiency needed for training. In fact, in terms of computational time, 1D and 2D datasets require only ∼5min and ∼1day for completing the training of each CNN respectively, compared to ∼3days for corresponding 3D ones given our computing resources availability mentioned in *Methods*. This difference is due to the larger number of weights that need to be estimated when training the network (3D model: 3,026, 2D model: 1,874, 1D model: 1,586).

However, the same performances were not preserved when the networks were applied out-of-sample. This is particularly evident for the 1D case, for which CNN accuracy dropped below usable level even for the alignment test. Average values of losses were variable when testing the 1D models, suggesting that the high uncertainty might be due to data overfitted during training. Similar to our results, other studies have also tried to compare 2D and 3D models, with the latter also performing better and returning a lower Mean Absolute Error (2D model: 40.5 ± 5.4HU; 3D model: 37.6 ± 5.1HU) [49].

### Limitations and strengths

This study presents several limitations. Our datasets are small compared to those commonly used for training DL models (e.g. CIFAR-10 dataset including >50,000 images [50]), and as a result, overfitting might have occurred during training [51]. However, it is hard to find larger FDOPA PET datasets than the one we used in this study, considering that we employed more than 350 real FDOPA brain PET scans, with an average cost of £5,000 each. Additionally, poor SNR maps used for the CNN2 training were all simulated. In fact, all the FDOPA PET data used in this study were acquired with full dose (>100 MBq) and no low dose scan was available. To simulate poor quality maps, Gaussian noise with zero mean and SD equal to 20% was added to the original images, to simulate the degradation of medical images deriving from photon, electronics and quantisation [52]. This is suboptimal as it might have introduced a simulation bias. More realistic noisy maps derived from subsampling of counts of existing measured data or phantoms should have been preferred. In particular, the use of a digital phantom ^53^ for both training and testing of the proposed CNN method were considered. However, real measured data were preferred since they represent scenario where the CNNs need ultimately to perform.

Another limitation of this work regards the type of data used for the QC testing. Rather than using raw FDOPA PET data, all our FDOPA maps were fully pre-processed and obtained with the same experimental design. Moreover, the approach used in this study was limited to FDOPA PET imaging in mental health applications and our datasets did not include maps from patients with tumours or obvious brain lesions. Future works should be extended to include neurological (e.g. PD patients) and oncological (e.g. glioma) cases. Nonetheless, the same DL approach could be extended to these cases and even to other PET tracer without any significant variations.

In terms of the analysis on data dimensionality reduction, the slice and line (used to derive the 2D and 1D models, respectively) were selected to maximise the striatal signal. However, this selection was arbitrary, and a different selection of slice/line might have led to different results. Future works should investigate the effect of different slice/line selection for 2D and 1D models, although we hypothesise that subsets of brain voxels with no striatal signal would be less informative than those we tested.

Lastly, this work considered only one type of DL CNNs. DenseNet was chosen as it has returned high performances in literature for other imaging applications [34], but alternative DL algorithms could be applied to the same problem. Future work should investigate different deep learning algorithms for automated neuroimaging QC, including a sensitivity analysis of method performances based on network parameters and their identification. Our preliminary tests on the CNN mostly used optimisers (e.g. Adam vs SGD) have shown that final performances of the models depend on them.

These tests might give new insights of DL results replicability.

## Conclusion

This proof-of-concept study has shown that deep learning convolutional neural networks could be used to perform automated QC of FDOPA PET imaging with promising performances when testing in independent datasets different from training samples. This work is relevant because it provides a framework to systematically and consistently assess large FDOPA PET datasets, without introducing operator-dependent bias.

Further studies need to be done to determine the generalisability of the methodology to different PET tracers, more heterogeneous patient population and to less processed data.

## Funding

This study was funded by Medical Research Council-UK (no. MC_U120097115), Maudsley Charity (no. 666), Brain and Behavior Research Foundation, and Wellcome Trust (no. 094849/Z/10/Z) grants to Dr Howes and the National Institute for Health Research (NIHR) Biomedical Research Centre at South London and Maudsley NHS Foundation Trust and King's College London. The views expressed are those of the author(s) and not necessarily those of the NHS, the NIHR or the Department of Health.

Dr Veronese is funded by the National Institute for Health Research Biomedical Research Centre at South London and Maudsley National Health Service Foundation Trust and King's College London, and by the Wellcome Trust Digital Award 215747/Z/19/Z. Dr Howes is funded by Medical Research Council grant MC- A656-5QD30, Maudsley Charity grant 666, support from the US Brain & Behavior Research Foundation, and Wellcome Trust grant 094849/Z/10/Z to Dr Howes and the National Institute for Health Research Biomedical Research Centre at South London and Maudsley National Health Service Foundation Trust and King's College London. Dr Jauhar is funded by the National Institute for Health Research Biomedical Research Centre at South London and Maudsley National Health Service Foundation Trust and King's College London, and a JMAS SIM Fellowship from the Royal College of Physicians, Edinburgh. Dr Bonoldi is supported by the National Institute for Health Research Biomedical Research Centre at South London, Maudsley National Health Service Foundation Trust, and King's College London.

## Potential conflicts of interest

Dr Howes is a part-time employee of H. Lundbeck A/S and has received investigator-initiated research funding from and/or participated in advisory/ speaker meetings organised by Angellini, Autifony, Biogen, Boehringer-Ingelheim, Eli Lilly, Heptares, Global Medical Education, Invicro, Jansenn, Lundbeck, Neurocrine, Otsuka, Sunovion, Rand, Recordati, Roche and Viatris/ Mylan. Neither Dr Howes or his family have holdings/ a financial stake in any pharmaceutical company. Dr Howes has a patent for the use of dopaminergic imaging.
